# Efflux Might Participate in Decreased Susceptibility to Oxytetracycline in Contagious Agalactia-Causative *Mycoplasma* spp.

**DOI:** 10.3390/ani11082449

**Published:** 2021-08-20

**Authors:** Juan Tatay-Dualde, Miranda Prats-van der Ham, Patrice Gaurivaud, Christian de la Fe, Florence Tardy

**Affiliations:** 1 Ruminant Health Research Group, Faculty of Veterinary Sciences, Regional Campus of International Excellence “Campus Mare Nostrum”, Campus de Espinardo s/n, University of Murcia, 30100 Murcia, Spain; juan.tatay@hotmail.com (J.T.-D.); miranda.prats@hotmail.es (M.P.-v.d.H.); cdelafe@um.es (C.d.l.F.); 2 UMR Mycoplasmoses Animales, Anses, VetAgro Sup, Université de Lyon, F-69364 Lyon, France; patrice.gaurivaud@anses.fr

**Keywords:** efflux pumps, antimicrobial resistance, mycoplasma, tetracyclines

## Abstract

**Simple Summary:**

Contagious agalactia is a multi-faceted disease affecting small ruminants worldwide. It is caused by four different *Mycoplasma* (sub)-species. In the absence of highly efficient vaccines, its control relies mostly on antibiotic treatment. Tetracyclines are one of the main families used, as they are cheap and often ensure clinical recovery, if not microbial clearance. However, some isolates have shown lowered susceptibilities even without the mutations in the target gene known to result in resistance. We suspected that an active efflux mechanism could be responsible for such lower-susceptibility phenotypes. Using various techniques, we demonstrated that most of the strains we studied did exhibit a capacity to actively extrude various substances including tetracyclines. This might contribute to low resistance profiles.

**Abstract:**

Contagious agalactia is associated with mastitis, keratoconjunctivitis, arthritis, pneumonia, and septicemia in small ruminants in countries with large dairy industries worldwide. The causative agents belong to four (sub)species of the *Mycoplasma* genus that have remained essentially susceptible to antimicrobials, including to the widely-used tetracycline family. However, some clinical isolates have been detected that show increased minimum inhibitory concentrations of tetracyclines, although they do not harbor the mutation in the 16SrRNA gene usually associated with resistance. The present work aimed to assess whether efflux pumps, infrequently described in mycoplasmas, could participate in the observed moderate loss of susceptibility. General efflux mechanisms were measured (i) using the fluorescence property of ethidium bromide when accumulated intracellularly and intercalated in the mycoplasma genomes, its active extrusion resulting in a temperature-dependent decrease in fluorescence and (ii) monitoring the growth inhibition of mycoplasmas by subinhibitory concentrations of tetracycline with or without reserpine, a known inhibitor of efflux in other bacteria. Both methods revealed non-specific efflux phenomena in most of the isolates tested, although their efficacy was difficult to quantify. This property could contribute to the acquisition of mutations conferring resistance by maintaining intracellular concentrations of tetracyclines at subinhibitory levels.

## 1. Introduction

Contagious agalactia (CA) is an infectious syndrome affecting small ruminants worldwide and especially countries in Southern Europe with large dairy industries. Its main clinical signs are mastitis, keratoconjunctivitis, and arthritis, but others such as pneumonia and septicemia have also been reported [1]. The negative economic impact of CA comes essentially from milk production loss and morbidity in young animals [2]. CA can be caused by four different *Mycoplasma* (sub)species: *M. agalactiae (Ma)*, the primary causative agent isolated from Spanish sheep and goat herds [3], and *M. mycoides* subsp. *capri (Mmc)*, *M. capricolum* subsp. *capricolum* *(Mcc),* and *M. putrefaciens (Mput)*, which belong or are related to the *M. mycoides* cluster [4] and are seldom isolated from sheep. *Mmc* is the main etiological agent responsible for clinical caprine CA in France [5,6].

There are no commercially available vaccines with demonstrated efficacy [7], and thus controlling CA mainly relies on biosafety strategies (culling or isolation of infected animals, hygiene measures, etc.) and chemotherapy [8]. Among the few antimicrobials with marketing authorization for small ruminants, the convenient broad-spectrum, low-cost tetracyclines are often used [9]. The activity of oxytetracycline against CA-causing mycoplasmas has been demonstrated in vitro in several studies, but isolates with increased minimal inhibitory concentration (MIC) suggest emerging resistance in the field [6,8,10,11,12,13,14].

In bacteria, resistance to tetracyclines has been associated with (i) ribosomal protection proteins, (ii) target mutation corresponding to mutations in the 16S rRNA (*rrs*) genes, (iii) efflux, or (iv) drug enzymatic inactivation [15,16]. Tet(M)-related ribosomal protection was shown to result in high tetracycline MIC values (MIC ≥ 8 µg/mL) in two human mycoplasmas, namely *M. hominis* and *Ureaplasma* spp. [17,18,19], but this mechanism has never been evidenced in animal mycoplasmas [20]. In the bovine pathogen *Mycoplasma bovis*, tetracycline resistance was correlated to hot-spot mutations in the 16S rRNA encoding genes [21,22]. In its closely related counterparts in small ruminants, *M. agalactiae*, some strains with decreased susceptibility did not harbor any significant binding site alterations [10]. Efflux pumps able to extrude tetracyclines were firstly characterized in *Escherichia coli* in 1980 [23] when more than 30 tetracycline pumps have been described in Gram-negative and -positive bacteria [15,16]. However, studies assessing efflux pumps in *Mycoplasma* spp. are scant and focus mainly on fluoroquinolones [24,25,26] or macrolides [27]. There are no previous reports on tetracycline efflux in *Mycoplasma* spp.

In a previous study, we evidenced that MICs at up to 4 µg/mL in different CA-associated clinical isolates were not always correlated with the presence of hot-spot mutations in the *rrs* genes, or mutations in the *rsp*J gene (30S ribosomal subunit protein S10) [10]. The aim of the present work was to assess whether efflux pumps could participate in the observed moderate loss of susceptibility. General efflux mechanisms were first measured indirectly using the fluorescence property of ethidium bromide (EtBr) intercalated between the bases of the genomic DNA. EtBr is known to (i) cross the cytoplasmic membrane in bacteria, (ii) accumulate intracellularly, generating an EtBr-polynucleotide fluorescent complex, and (iii) in cases of resistance, be extruded by both proton-motive force and ATP-dependent systems [28]. The efflux was then assessed by monitoring the growth inhibition of *Mycoplasma* spp. by tetracycline at subinhibitory concentrations in the presence or absence of reserpine, a plant-derived alkaloid known to inhibit tetracycline efflux in other bacterial models [29]. Both methods demonstrated the existence of an efflux, but its quantification in different isolates was difficult to standardize.

## 2. Materials and Methods

### 2.1. Mycoplasma Isolates

A total of 32 *Mycoplasma* spp. isolates were collected from contagious agalactia outbreaks (mastitis, arthritis or pneumonia) or from bulk tank milk during inspections in France and Spain (Table 1). They comprised 17 *M. agalactiae* and 15 strains of the so-called *M. mycoides* cluster or related (5 *Mmc*, 4 *Mcc,* and 6 *Mput*). Their identification, oxytetracycline MICs (from 0.25 to 8 µg/mL) and their corresponding 16S rRNA and *rpsJ* genotypes were characterized in a previous study [10].

### 2.2. Efflux Assay by EtBr (EtBr)–Agar Method

This agar-based method relies on the ability of EtBr to cross the cytoplasmic membrane, accumulate in mycoplasma cells, and fluoresce under UV light when intercalated in DNA. Active efflux systems result in a temperature-dependent depletion of intracellular EtBr and hence a decrease in the fluorescence. Our assays were adapted from the work of Martins et al. on other bacterial models with some modifications [30,31]. Briefly, two-fold EtBr dilutions ranging from 0.2 to 6.4 µg/mL were prepared in PPLO–agar (Indicia Production, St Genis L’Argentière, France). Plates were inoculated with spots of 1 µL of each isolate at 10^7^–10^8^ cfu/mL for *M. agalactiae* and 10^6^–10^7^ cfu/mL for *M. mycoides* cluster or related strains, and were incubated at 37 °C with 5% CO_2_ for 72 h to obtain confluent colonies. One set of plates (Set 1) was then incubated in the same conditions for another 24 h. The other set (Set 2) was stored at 4 °C. After the total 96 h incubation, fluorescence was examined under UV light using a transilluminator (Gel Doc^TM^ XR System, Bio-Rad, Marnes-la-Coquette, France), with an exposure time of 1 s, and plates were photographed.

### 2.3. Monitoring of EtBr Efflux by Fluorometry

This technique was adapted from previously described protocols for other bacteria or other mycoplasmal species [26,32,33]. Briefly, mycoplasma isolates were grown in 2 mL of PPLO broth (Indicia, France) until they reached the stationary phase (~10^9^ cfu/mL), centrifuged to obtain a cell pellet (10,000× *g*, 20 min, 20 °C) and resuspended in 2 mL of PBS 1X with EtBr at 10 µg/mL and reserpine at 20 µg/mL to inhibit potential efflux. These cells were then incubated at 37 °C for 30 min with agitation to facilitate the uptake of EtBr. Strains were then centrifuged again (10,000× *g*, 15 min, 20 °C) and the supernatant was discarded. Cells were resuspended in 1 mL (for *M. agalactiae* strains) or 2 mL (for *M. mycoides* cluster or related strains) of PBS 1X. For each condition studied, 200 µL of this cell suspension were placed in a well of a Greiner black, flat-bottom, 96-well plate (Sigma-Aldrich, Saint-Quentin-Fallavier, France) to measure fluorescence every minute for 1 h. Bacterial efflux activity was assessed in different conditions: (i) with no energy source (only PBS 1X), (ii) adding energizers such as pyruvate (0.5% *m*/V final concentration) or glucose (0.5% *m*/V final concentration), and (iii) at 37 °C versus 25 °C to reduce mycoplasma metabolism. Measurements were made using the CLARIOstar^®^ plate reader (BMG LABTECH, Champigny s/Marne, France) with excitation and emission wavelengths of 360 nm and 590 nm, respectively, as recommended by the EtBr supplier (Sigma-Aldrich, Saint-Quentin-Fallavier, France). Focal height and gain were automatically adjusted before initiating the reading process. The maximal fluorescence activity reached after EtBr uptake was normalized to 1 (i.e., 100%). Energizers were added after the first 5 min and at the end of the experiment (60 min), and SDS (Sodium Dodecyl Sulfate, 1% *m*/V final concentration in each well) was added to lyse cells and release the remaining EtBr. Analyses were repeated three times for each strain and condition.

### 2.4. Growth in the Presence of Subinhibitory Concentrations of Oxytetracycline

Ten isolates (5 *M. agalactiae*, 3 *Mmc* and 2 *Mcc*) were grown in the presence of subinhibitory concentrations of oxytetracycline (one fourth of their MIC) with or without reserpine (20 µg/mL) at 37 °C using a protocol adapted from Kovacevic et al. [34]. Briefly, mycoplasma cells were inoculated at 10^4^ cfu/mL in PPLO broth supplemented with 0.5% (*m*/V) pyruvate in different wells of a sterile 96-well plate (final volume in each well 200 μL). When necessary, reserpine and/or oxytetracycline were added. The effect of reserpine alone on bacterial growth was also assessed. The plates were sealed and incubated with agitation at 37 °C. Colony counts were performed at *t* = 0, 16, 24, 40, 48, 64, 72, 88, and 96 h of incubation using a multi sample inoculator to deposit 1 μL from each well onto an agar plate (Mast Uri^®^ Dot, Mast Diagnostic, Amiens, France). To obtain countable values, 1/100 and 1/20,000 dilutions were made in sterile PBS 1X. Each experiment was repeated twice.

## 3. Results

### 3.1. Efflux Assay by EtBr (EtBr)–Agar Method

**Figure 1:**
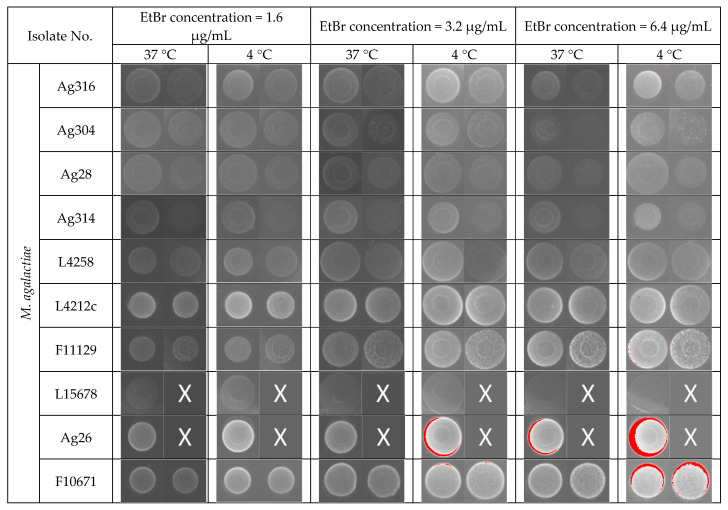

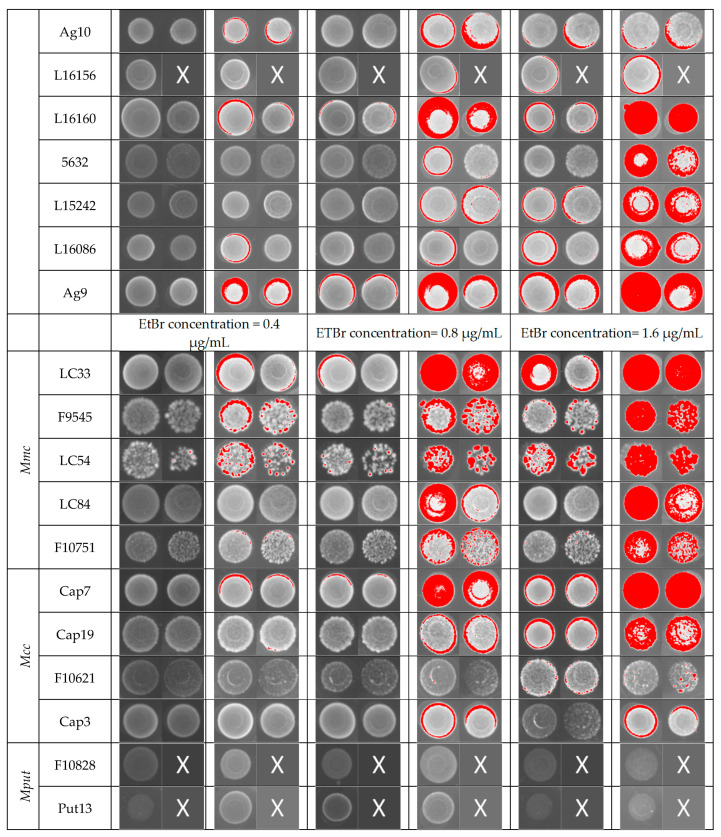
Evaluation of efflux activity of *Mycoplasma* isolates using the ethidium bromide (EtBr) agar method. A volume of 1 µL of each culture dilution (10^8^ and 10^7^ cfu/mL for *M. agalactiae* and 10^7^ and 10^6^ cfu/mL for *M. mycoides* cluster, or related, isolates) was incubated for 72 h at 37 °C and a further 24 h at either 37 °C or 4 °C on agar plates containing different EtBr concentrations. Fluorescence was detected under UV light (the appearance of pixel saturation resulted in a red coloration of bacterial spots). “X” means that only one concentration of inoculum was studied (10^8^ cfu/mL for *M. agalactiae* and 10^7^ cfu/mL for *M. mycoides* cluster, or related strains). *Mmc*, *M. mycoides* susbp. *capri*; *Mcc*, *M. capricolum* subsp. *capricolum*, *Mput*., *M. putrefaciens*.

Figure 1 illustrates the different patterns of colonies fluorescence using increasing concentrations of EtBr and different incubation schemes. Some isolates of *Mput*, not shown in Figure 1, were inhibited at EtBr concentrations of 1.6 μg/mL, suggesting the MIC was reached, and were not further analyzed (noted “inhibited” in Table 1). The appearance of pixel saturation resulted in a red coloration of bacterial spots. This saturation increased as expected with increased concentrations of EtBr. In the absence of red saturation for 8 *M. agalactiae* and 2 *Mput* strains, the fluorescence was not interpreted (n.i., not interpreted in Table 1), as the white fluorescence was difficult to distinguish from the bacterial spot background. Most of the *Mmc* and *Mcc* strains showed saturated colonies at 1.6 µg/mL of EtBr at 37 °C, whereas the *M. agalactiae* strains did so at 6.4 µg/mL except for strain L16160 and Ag9 (3.2 µg/mL). This could result from a better EtBr intake capacity and/or a lower efflux for *Mcc* and *Mmc* strains. For all these strains, the fluorescence level (read as the relative proportion of saturated pixels) was compared between plates transferred to 4 °C and those left at 37 °C, after a 72 h-intracellular accumulation of EtBr at 37 °C. The 9 *M. agalactiae* isolates with saturated pixels were divided into two groups: one with a moderate almost equivalent saturation at 4 °C and 37 °C at 6.4 µg/mL of EtBr (Ag26, F10671, Ag10, L16156) and one with a markedly high saturation at 6.4 µg/mL of EtBr at 4 °C, but not at 37 °C (L16160, 5632, L15242, L16086, Ag9, noted as “+++” in Table 1). For most of the *Mcc* and *Mmc* strains, except for strains *Mcc* F10261 and cap3, the efflux was blocked at 4 °C, resulting in a saturation of fluorescence as low as 0.4 µg/mL.

This suggests that most but not all of the strains express temperature-dependent systems capable of extruding EtBr [30,31]. A semiquantitative evaluation of the efflux system efficacy was done based on both the EtBr concentrations resulting in saturation at 37 °C (the higher the concentration, the more efficient the system) and the intensity difference between 4 °C and 37 °C (the greater the difference, the more efficient the efflux) (Table 1).

### 3.2. Monitoring of EtBr Efflux by Fluorometry

**Figure 2:**
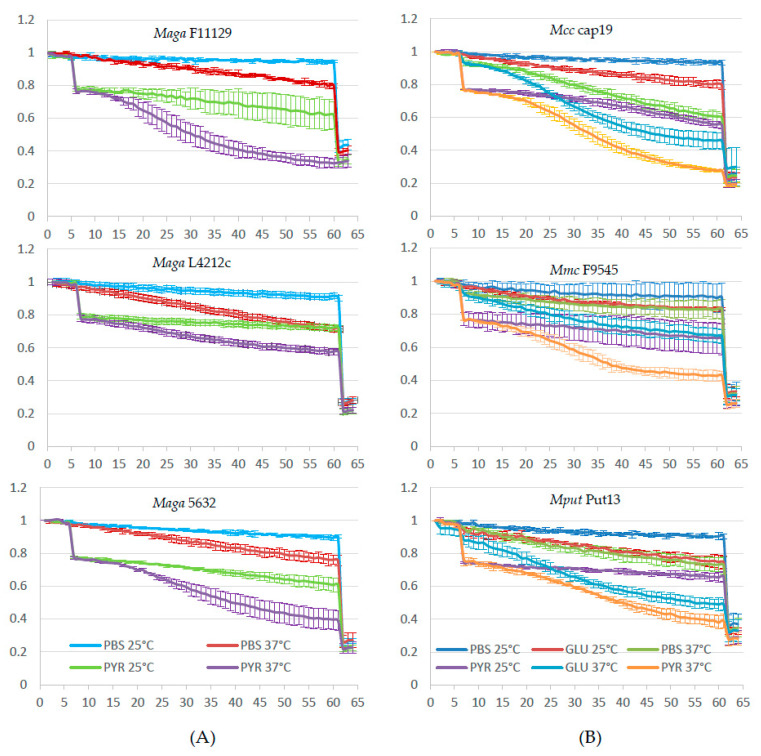
Determination of ethidium bromide efflux by fluorometry in cell suspensions of *M. agalactiae* (**A**) and *M. mycoides* cluster or related (*Mcc*, *M. capricolum* subsp. *capricolum*; *Mmc*, *M. mycoides* subsp. *capri*; *Mput*, *M. putrefaciens*) (**B**) strains. Experiments were conducted at 25 °C or 37 °C, with or without the addition of an energy source (pyruvate, PYR or glucose, GLU) at *t* = 5 min. The complete release of EtBr was obtained by addition of SDS (1 %) at *t* = 60 min. *x*-axis, time in minutes; *y*-axis, mean relative fluorescence (of three repeated measures, with standard deviations).

To quantify EtBr efflux more accurately, a real-time detection method was developed to monitor the loss of fluorescence of EtBr previously accumulated in *Mycoplasma* cells and bound to DNA and RNA. The effect of the incubation temperature (25 °C versus 37 °C) and presence of energizers (glucose or pyruvate) was also tested. Figure 2 illustrates the changes in fluorescence in each of the studied conditions for six representative strains. Supplementary Figure S1 (*M. agalactiae*) and Figure S2 (*M. mycoides* susbp. *capri*; *M. capricolum* subsp. *capricolum* and *M. putrefaciens*) give the results obtained for all the isolates studied. The maximum intensity reached at the beginning of the experiment was normalized to 1. In the absence of any energizer, the fluorescence was shown to decrease to [0.66–0.83] at 37 °C and only to [0.84–0.96] at 25 °C, which could result from temperature-dependent diffusion or low-level efflux (Figure 2). Adding an energizer resulted in an immediate drop in fluorescence, more marked with pyruvate than with glucose (tested only for subspecies of, or related to, the cluster *M. mycoides*), followed by a slower fluorescence decrease to reach [0.28–0.66] at 37 °C and [0.52–0.72] at 25 °C after 60 min, with large differences between the different isolates (Figure 2), indicating the presence of temperature-dependent efflux pumps. Interestingly, when available, the kinetics for fluorescence decrease were roughly parallel in the presence of pyruvate and glucose (e.g., see strain *Mcc* cap19 or *Mput* Put13), suggesting a similar mode of action for these two energizers.

The final addition at *t* = 60 min of SDS released the remaining intracellular EtBr and reduced fluorescence to [0.2–0.3] for all the strains studied. The efflux capacity observed in the presence of an energizer varied widely between strains. An attempt was made to convert the observed kinetics into semiquantitative data for comparison between strains (Table 1). The total, i.e., immediate and slower, drop in fluorescence after adding the energizer, was considered. It was calculated by subtracting the residual fluorescence after 60 min in the presence of the energizers from the fluorescence at *t* = 60 min with PBS alone, at both 25 °C and 37 °C. Most of the strains were shown to be able to actively extrude EtBr, more efficiently at 37 °C than at 25 °C and in the presence of an energizer, and for isolates of the *M. mycoides* cluster, the maximum drop in fluorescence was obtained with pyruvate at 37 °C. In this condition, the maximum drop in fluorescence ranged between −0.14 and −0.52. For instance, *Mcc* Cap19, F10261 or *M. agalactiae* F11129, Ag28 and Ag304 were able to expel almost 100% of EtBr at 37 °C in the presence of pyruvate, and the addition of SDS resulted only in a slight further drop in fluorescence. By contrast, *M. agalactiae* L4212c and Ag26 had poor efflux capacity: half of the fluorescence was still present after 60 min at 37 °C in the presence of pyruvate. No poor efflux capacity was observed within strains of the *M. mycoides* cluster.

However, these observations did not correlate well with the categorization obtained using the EtBr–agar method (Table 1). *M. agalactiae* strains with an intermediate (++) or high (+++) efflux activity on agar screening showed a mean loss of fluorescence of 0.38 +/− 0.02 (*n* = 5, [0.34–0.40]) while those ranked (+) showed a very variable loss, with a mean of 0.31 +/− 0.11 (*n* = 4, [0.18–0.42]). A comparison was also performed for uninterpretable strains, for instance with strain Ag28, which did not become fluorescent in any of the conditions tested in the EtBr–agar method, yet showed the highest active efflux activity of the studied *M. agalactiae* strains (Figure 1 and Table 1). Likewise, *Mcc* and *Mmc* strains, with an intermediate (++) or high (+++) efflux activity, showed a loss of fluorescence of 0.40 +/− 0.06 (*n* = 7, [0.34–0.52]) not different from strains ranked (+) or (+/−) (0.39 and 0. 48 respectively). EtBr-based methods thus demonstrated a general efflux capacity, temperature sensitive and energy-driven, that varies among strains, albeit more universal within the cluster *M. mycoides*, but in our hands, they did not adequately quantify this efflux.

### 3.3. Growth in the Presence of Subinhibitory Concentrations of Oxytetracycline

**Figure 3:**
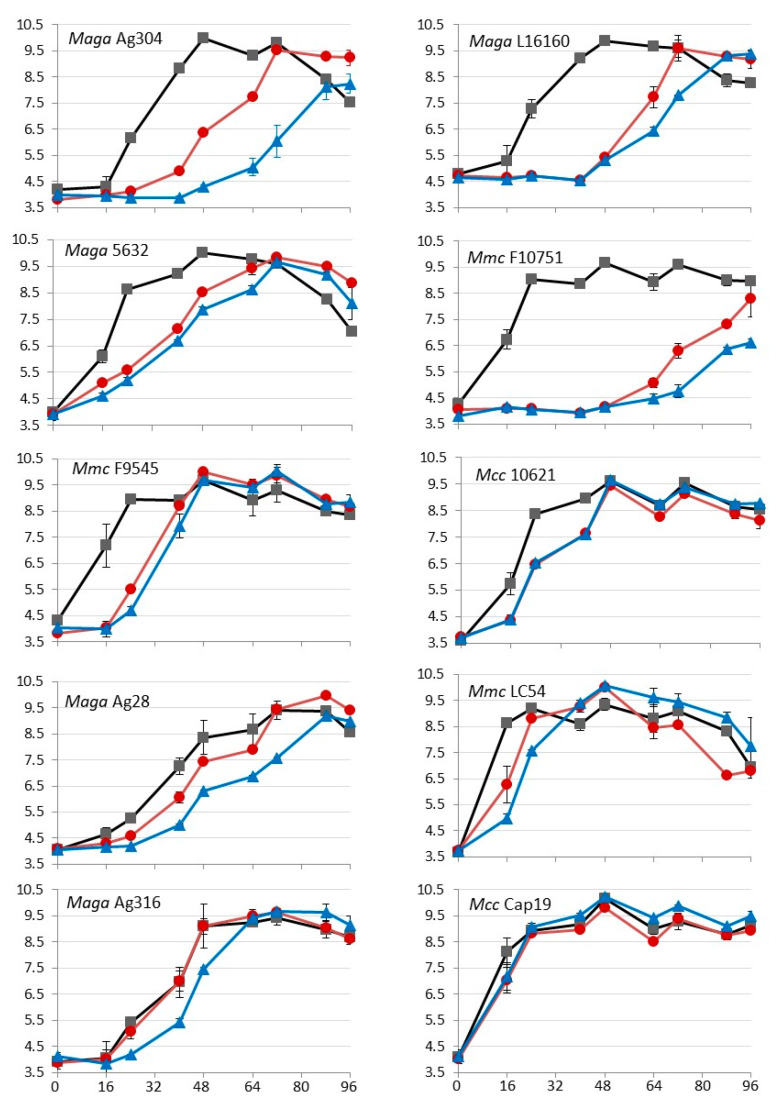
Growth kinetics of *M. mycoides* subsp. *capri*, *M. capricolum* subsp. *Capricolum,* and *M. agalactiae* strains obtained under different culture conditions: broth medium only (black curves), broth medium with oxytetracycline (red curves), and broth medium with oxytetracycline and reserpine at 20 μg/mL (blue curves). The specific concentrations of oxytetracyline corresponded to ¼ of the MIC per strain (i.e., 0.0625 µg/mL for *Mcc* Cap19; 0.5 µg/mL for *Maga* Ag204, *Maga* 5632, *Mcc* 10621, *Mmc* LC54; 1 µg/mL for *Maga* L16160, *Mmc* F10751, *Mmc* F9545, *Maga* Ag28 and 2 µg/mL for *Maga* Ag316). Reserpine was added at 20 µg/mL (blue curves); *x*-axis: incubation time (in hours) and *y*-axis: bacterial counts (in log10 cfu/mL).

This part of the study was designed to assess whether the general efflux observed with EtBr was also able to extrude oxytetracycline and could contribute to increased MIC values (Table 1). Nine isolates with increased oxytetracycline MICs of 2, 4, and 8 µg/mL, respectively, and with intermediate (++) or high (+++) EtBr efflux were selected. Strain *Mcc* Cap19 was also added as a control, because it showed high EtBr efflux capacity but had a low MIC of oxytetracycline (0.25 µg/mL). These isolates were grown in the presence of oxytetracycline at a concentration corresponding to ¼ of the MIC with or without reserpine (20 µg/mL). The growth inhibition was compared to the growth in PPLO medium alone. In an independent experiment, we verified that reserpine (20 μg/mL) alone had no effect on mycoplasma growth (Figure 3). The bacteriostatic effect of oxytetracycline at ¼ of the MIC was either marked, with a least two time points showing a log2 delay in cfu/mL (*Ma* Ag304, *Ma* L16160, *Ma* 5632, *Mmc* F10751 and *Mmc* F9545), or moderate (*Mcc* 10621, Ma Ag28, *Mmc* LC54) to nil (*Mmc* LC54, *Ma* Ag316, *Mcc* cap19). In experimental conditions when growth inhibition by oxytetracyline failed, the presence of reserpine had either no effect (*Mcc* cap19, which was the low MIC control) or a potential enhancer effect on the bacteriostatic property of tetracycline (AG 316), suggesting that an efflux was responsible for the absence of the oxytetracyline effect. When a growth inhibition by oxytetracycline was observed, two responses to reserpine were noted: (i) the growth inhibition remained unchanged (*Mcc* 10621, *Maga* 5632, *Mmc* 9545) or (ii) it was clearly enhanced (at least two time points with one log difference) (Ag 304, Ag 28, Mmc LC54), sometimes only after 48 h of growth (Ag16160, Mmc 10751).

This demonstrates that the inhibition of efflux pumps clearly potentiated the bacteriostatic effect of oxytetracycline, which was stronger and longer-lasting in the presence of reserpine. Semiquantitative marks (Table 1) were assigned by dividing the maximum difference observed between the growth curves obtained with and without reserpine for Ag304 (3.5 log10) to delimit four levels of inhibition, ranging from less than 0.9 log10 (+/−) to more than 2.6 log10 (+++). The efflux capacities estimated using EtBr fluorescence and using growth inhibition kinetics were comparable for *M. agalactiae* strains Ag304, Ag28, L16160, Ag316, and for *Mmc* strains LC54 and F10751, but not for strains *Ma* 5632, *Mmc* F9545, and *Mcc* 10621. For the low susceptibility control (*Mcc* cap19), the results were not interpreted, because the oxytetracycline concentration used was potentially too low (0.0625 µg/mL at ¼ of the MIC) to observe any inhibition.

## 4. Discussion

In a previous study, we observed a moderate decrease in susceptibility to oxytetracycline in some *Mycoplasma* strains belonging to species involved in contagious agalactia syndrome of small ruminants, *M. agalactiae*, *Mmc* and *Mcc* [10]. Only *Mput* did not show any in vitro increased tetracycline MICs, which was recently confirmed [6]. Three main mechanisms, namely target protection, target modification, and efflux extrusion, are known to result in tetracycline resistance in bacteria. Target protection has so far never been observed in animal mycoplasmas [20]. Tetracycline resistance associated with target mutations in the 16S rRNA genes and in the *rps*J gene coding for the ribosomal protein S10 is well described for mycoplasma of animal origin, and such mutations were observed for field isolates of *M. agalactiae*, *Mmc* and *Mcc* harboring a MIC of oxytetracycline ranging from 2 to 8 µg/mL [10]. However, several isolates with similar increased tetracycline MIC showed no mutation in the *rrs* and/or *rpsJ* genes. We hypothesized that efflux mechanisms could be involved in the loss of tetracycline susceptibility for these specific isolates [10].

Bacterial efflux pumps are classified into five families: the adenosine triphosphate (ATP)-binding cassette (ABC) superfamily, the resistance-nodulation-division (RND) family, the small multidrug resistance (SMR) family, the major facilitator superfamily (MFS), and the multidrug and toxic compound extrusion (MATE) family [35]. Four of them have been described as able to extrude tetracycline (SMR, MFS, RND and ABC families). Efflux in mycoplasma has seldom been evidenced except for fluoroquinolones in *M. hominis* [26] and in *Mmc* [24], and in *M. pneumoniae* for macrolides [27], all three studies pointing to an ABC-type efflux pump, able to also extrude unrelated compounds such as EtBr. A multi-drug efflux transporter of the MATE family has also been suggested in *M. bovis* though not formally demonstrated [36]. In general, efflux pumps in bacteria confer cross-resistance to a wide range of entities, including dyes, detergents, disinfectants, and antimicrobials [37]. Dyes such as EtBr are of particular interest as they can be easily used as markers of an efflux phenomenon. Other ways to identify efflux pumps include their inhibition and how it impacts on resistance to antimicrobials. The plant-derived alkaloid reserpine is known to inhibit both ATP binding cassette and major facilitator efflux transporters, although its exact mechanism of action is still unclear. Convergent results from different experiments are often necessary to demonstrate the presence of efflux pumps with certainty. For instance, in *Staphylococcus aureus*, the reserpine screen failed to identify overexpression of one or more MDR efflux pump genes, as shown by qRT-PCR [38].

In this study, we assessed the presence of an efflux in field isolates of mycoplasmas involved in CA syndrome using two different EtBr-based assays, together with a growth inhibition experiment with oxytetracycline in the presence or absence of reserpine. The EtBr agar plate method was first assessed as it allows a fast, easy screening of many bacterial isolates. However, for some of the *Mycoplasma* isolates tested, the results were not always interpretable owing to either a growth inhibition of mycoplasma colonies in the presence of EtBr in the agar medium (e.g., the presence of EtBr, at 0.4 µg/mL in the agar plate, resulted in a complete growth inhibition for 4 out of 6 *M. putrefaciens* isolates) or to a fluorescence too low to be readable. The last effect could result from (i) a partial growth inhibition limiting colony development and/or (ii) a default in the accumulation of EtBr within the cells, because *Mycoplasma* colonies were grown on a complete medium containing energy sources that could potentiate efflux systems in the first 72 h of incubation. The second hypothesis was supported as transfer of plates at 4 °C for a further 24 h incubation blocked the efflux, resulting in a higher fluorescence than their counterparts maintained et 37 °C. This phenomenon was marked for 12 isolates and was more moderate for 6 other isolates, whatever their species, suggesting a temperature-sensitive efflux system.

A more dynamic EtBr-based assay relying on efflux kinetics in PBS was also used. Unlike the agar plate method, no cell growth was expected in the presence of EtBr (as the contact time was only 30 min) and EtBr accumulation within the cells was maximized by using reserpine to inhibit efflux. This helped to harmonize the quantity of EtBr within the cells before starting the observation of the EtBr release time and thus the fluorescence decrease. With this method, all our isolates gave readable results and showed an efflux of EtBr energized by adding pyruvate, a known energy source for mycoplasmas. The efflux was slower when the temperature went from 37 °C to 25 °C, but it varied widely between strains within each species. We failed to establish a coherent link between the efflux level and the tetracycline MIC values. The clearest example was that of strain *M*. *capricolum* subsp. *capricolum* Cap19 with an oxytetracycline MIC of 0.25 µg/mL, but a marked EtBr efflux in both agar plates and the fluorometry assays (Figure 1 and Figure 2).

Taken together, the EtBr experiments evidenced efflux mechanisms in mycoplasma species involved in CA, with a high inter-strain variability, not correlated to the overall oxytetracyline susceptibility.

Potentiation by adding reserpine of growth inhibition due to oxytetracycline was assessed for nine representative isolates selected for their increased MICs (2–8 µg/mL) in comparison with a susceptible *Mcc* control isolate (*Mcc* cap19, MIC of 0.25 µg/mL). For three isolates, adding of reserpine had a low to nil effect on growth inhibition (*Mcc* 10621, *Maga* 5632, *Mmc* 9545). However, isolates *Ma* 5632 and *Mmc* 9545 showed a capacity to extrude EtBr, suggesting that there might be some specificity in the entities transported by the efflux pumps or that some pumps are not inhibited by reserpine. Of the six remaining isolates (4 *Ma* and 2 *Mmc*), reserpine enhanced the growth delay due to oxytetracycline, with different intensities between isolates and sometimes time-dependently (isolates Ag16160 and *Mmc* F10751). Interestingly, the effect of reserpine in our experimental conditions was maximum with isolate Ag304, with an MIC of 2 µg/mL and no *rrs* gene mutation. Approximately half the effect was observed for other isolates of MIC 2 to 8 µg/mL, with (Ag316, *Mmc* LC54, Ag28), or without (*Ma* 16160, *Mmc* 10751) *rrs* or *rpsJ* mutations. Our observations suggest that the efflux system in mycoplasma allows tetracycline resistance up to 4 µg/mL and might facilitate the acquisition of a target mutation for higher resistance. This is consistent with the fact that the M. *putrefaciens* species, which has remained so far mostly susceptible to tetracycline [6], exhibits no or a weak efflux. On the experimental basis proposed here, it would be interesting to further test the contribution of efflux in other *Mycoplasma* species with high tetracycline MICs and the corresponding mutations in the target genes, such as *M. bovis*.

Early studies suggested that without a rigid peptidoglycan-based cell wall or actin-based cytoskeleton structure, mycoplasma cells could regulate their cell volume and adapt to different osmotic environment through active solute extrusions [39]. The effect would be to osmotically balance the colloid osmotic effect and work against osmotic lysis. Glucose-enhanced pumps were notably described as extruding Na+ ions. Hence, the efflux might rely on a mechanism not specific to antimicrobials and very variable in expression between strains in time, depending on the nutritional environment. Its contribution to resistance in vivo, with very variable environments depending on the colonized body niche remains to be demonstrated.

## 5. Conclusions

We evidenced potential efflux mechanisms in mycoplasma species involved in CA, with a high inter-strain variability. The efflux efficacy was not always comparable for the ethidium bromide dye versus oxytetracycline and was not always correlated to oxytetracyline susceptibility, suggesting non-specific mechanisms. The contribution of efflux pumps to the general antimicrobial resistance in *Mycoplasma* spp. now needs to be studied.

## Figures and Tables

**Table 1:** The 32 mycoplasma isolates included in the study and details of their origin, oxytetracycline MICs, *rrs*/*rpsJ* genotype when available, and results obtained in the different experiments described in this work.

Isolates		Isolation Context			MIC (µg/mL)	Mutations ^2^		EtBr Efflux		Oxytetracycline Efflux ^5^
No.	Origin ^1^	Sample	Year	Host		*rrs*	*rpsJ*	Agar ^3^	Kinetics ^4^	
*M. agalactiae*										
L4258	FR-79	Mastitic milk	<1989	Goat	0.25	No	nd	n.i.	44 ± 6	nd
Ag314	ES-ZA	Bulk tank milk	2015	Sheep	0.5	No	nd	n.i.	44 ± 9	nd
F11129	FR-26	Bulk tank milk	2016	Goat	0.5	nd	nd	n.i.	47 ± 2	nd
L15678	FR-64	Bulk tank milk	2009	Sheep	0.5	No	nd	n.i.	39 ± 3	nd
L4212c	FR-84	Mastitic milk	1986	Sheep	0.5	nd	nd	n.i.	14 ± 2	nd
5632	ES-nk	Joint	<1991	Goat	2	nd	nd	+++	37 ± 4	+ (0.8)
Ag304	ES-MA	Mastitic milk	2015	Goat	2	No	nd	n.i.	45 ± 7	++ (3.5)
Ag9	ES-MU	Mastitic milk	2009	Goat	2	No	nd	+++	37 ± 1	nd
Ag26	ES-MU	Bulk tank milk	2000	Goat	2	No	nd	+	18 ± 3	nd
Ag10	ES-MU	Mastitic milk	2008	Goat	2	No	nd	+	27 ± 11	nd
F10671	FR-16	Mastitic milk	2011	Goat	2	No	nd	+	39 ± 4	nd
L16086	FR-84	Mastitic milk	2013	Goat	2	No	nd	+++	38 ± 4	nd
L15242	FR-86	Mastitic milk	2009	Goat	2	No	nd	+++	40 ± 9	nd
Ag28	ES-MU	Bulk tank milk	2000	Goat	4	Yes	nd	n.i.	47 ± 10	++ (1.9)
L16160	FR-49	Bulk tank milk	2010	Goat	4	No	No	+++	38 ± 4	++ (1.8)
L16156	FR-79	Bulk tank milk	2010	Goat	4	No	No	+	40 ± 3	nd
Ag316	ES-AL	Mastitic milk	2015	Goat	8	Yes	No	n.i.	43 ± 7	++ (1.7)
*M. capricolum* subsp. *capricolum*										
Cap3	ES-AN	Mastitic milk	2011	Goat	0.25	No	nd	+	39 ± 1	nd
Cap7	ES-MA	Auricular swab	2009	Goat	0.25	nd	No	+++	34 ± 9	nd
Cap19	ES-GC	Mastitic milk	2015	Goat	0.25	nd	nd	+++	52 ± 2	− (0.0)
F10621	FR-79	Mastitic milk	2015	Goat	2	No	Yes	+/−	46 ± 2	− (0.1)
*M. mycoides* subsp. *capri*										
LC84	ES-GC	Mastitic milk	2014	Goat	0.5	No	nd	+++	41 ± 8	nd
LC33	ES-MU	Bulk tank milk	2009	Goat	0.5	No	nd	++	39 ± 4	nd
LC54	ES-GC	Bulk tank milk	2004	Goat	2	No	Yes	+++	35 ± 10	++ (1.5)
F9545	FR-81	Lung	2012	Goat	4	No	Yes	+++	40 ± 5	+ (0.9)
F10751	FR-79	Mastitic milk	2014	Goat	4	No	No	+++	36 ± 1	++ (2.2)
*M. putrefaciens*										
F10828	FR-74	Mastitic milk	2016	Goat	0.5	nd	nd	n.i.	34 ± 2	nd
Put13	ES-GC	Mastitic milk	2015	Goat	0.5	No	nd	n.i.	34 ± 2	nd
F5435	FR-36	Mastitic milk	2008	Goat	0.5	No	nd	Inh.	32 ± 3	nd
F8131	FR-23	Mastitic milk	2013	Goat	0.5	No	nd	Inh.	37 ± 5	nd
F1174	FR-79	Bulk tank milk	2004	Goat	0.5	nd	nd	Inh.	37 ± 3	nd
Put9	ES-GC	Mastitic milk	2014	Goat	0.5	nd	nd	Inh.	33 ± 4	nd

^1^ Origin is ES, Spain or FR, France. FR is followed by the department number (nk, not known) and ES by province names according to ISO codes as defined at https://www.iso.org/obp/ui/es/#iso:code:3166:ES (accessed on 19 August 2021). ^2^ Mutations affecting Helix31 of the Tet-1 binding site and ribosomal protein S10, previously associated with tetracycline resistance in these mycoplasma species, described by Prats-van der Ham et al., 2018 [10]. nd, not done. ^3^ Differences in ethidium bromide (EtBr) intracellular concentration between plates incubated at 37 °C and at 4 °C, as estimated by fluorescence and transformed into semiquantitative scores (see Section 3.1). n.i., not interpretable; Inh., inhibited. ^4^ Loss of fluorescence (%) due to ethidium bromide active efflux after 60 min with pyruvate (pyr) at 37 °C. ^5^ Growth delay (difference between microbial counts in log scale) due to efflux pump inhibition (comparing incubation with oxytetracycline and with and without reserpine) is represented semi-quantitatively (four levels of inhibition were defined, ranging from less than 0.9 log10 (+/−) to more than 2.6 log10 (+++)).

## Data Availability

The data presented in this study are available on request from the corresponding author.

## Supplementary Materials

The following are available online at https://www.mdpi.com/article/10.3390/ani11082449/s1, Figure S1: Determination of the ethidium bromide efflux by fluorometry in *M. agalactiae* isolates; Figure S2: Determination of the ethidium bromide efflux by fluorometry in *M. capricolum* subsp. *capricolum*; *M*. *mycoides* subsp. *capri* and *M. putrefaciens* isolates.
