# Supplementary figures and images for: Efflux Might Participate in Decreased Susceptibility to Oxytetracycline in Contagious Agalactia-Causative Mycoplasma spp
Source: Animals (Basel). 2021 Aug 20;11(8):2449. doi: 10.3390/ani11082449 (PMC8388784; doi:10.3390/ani11082449)

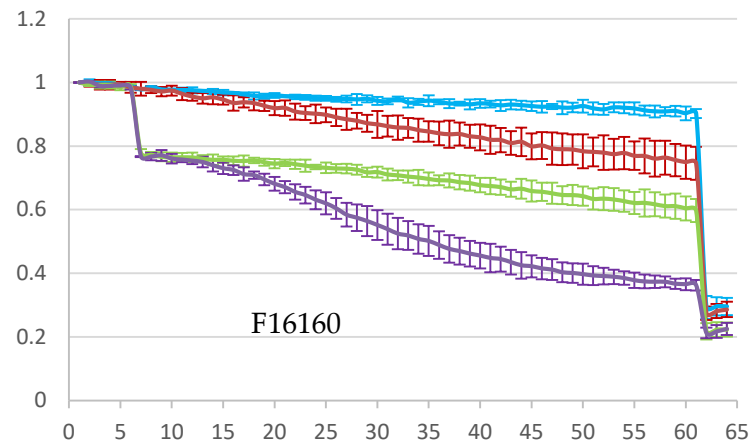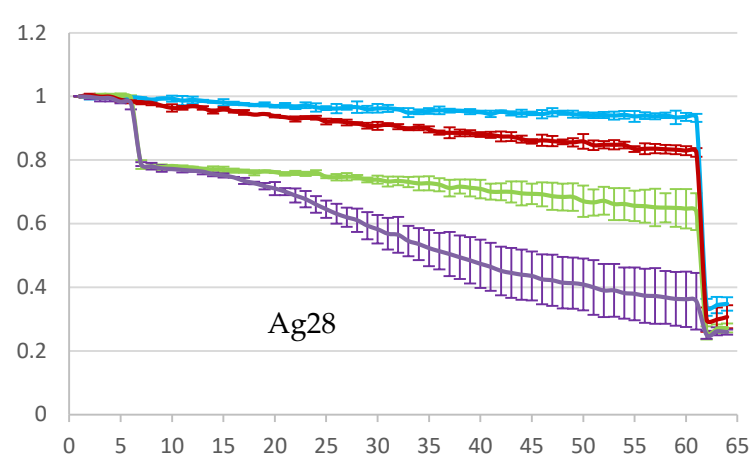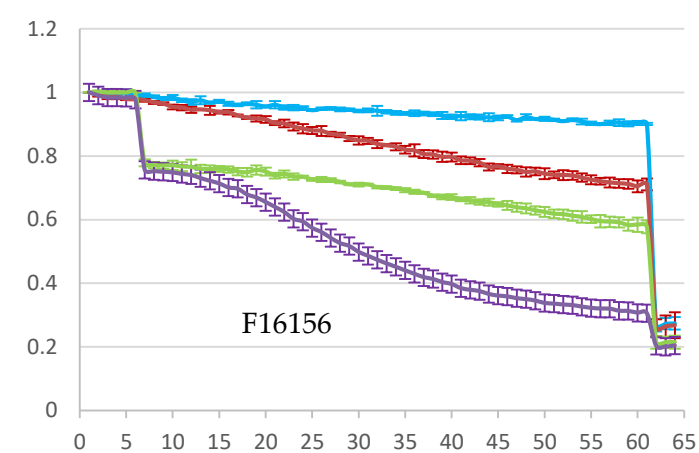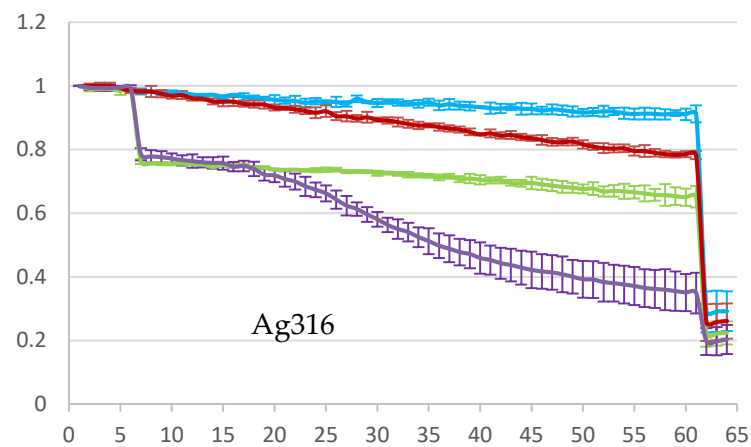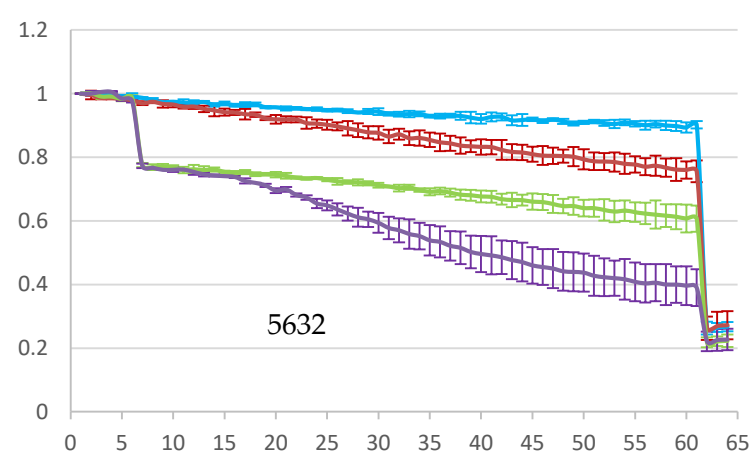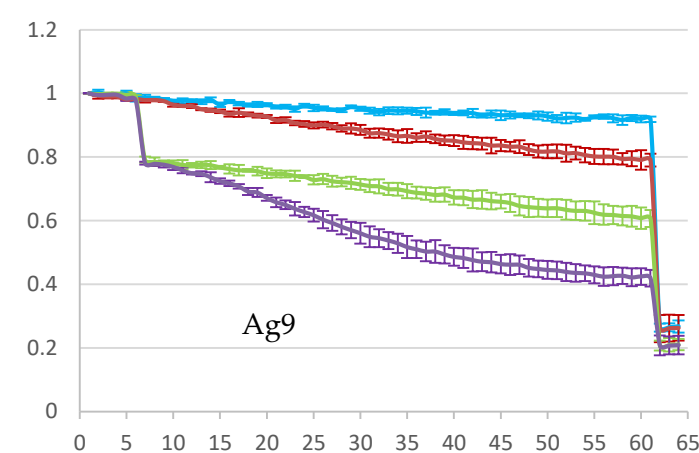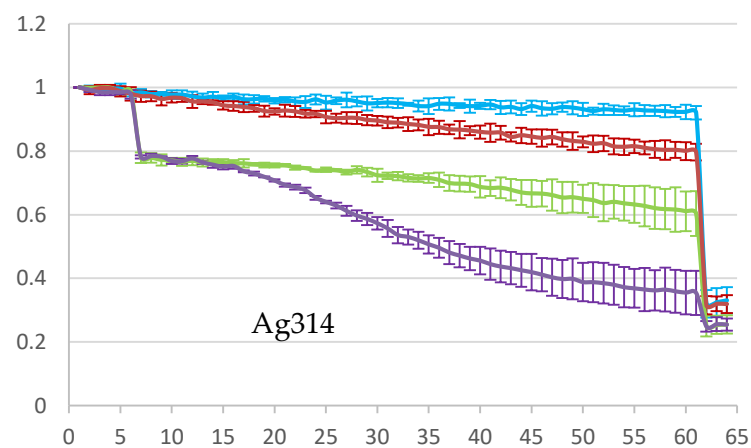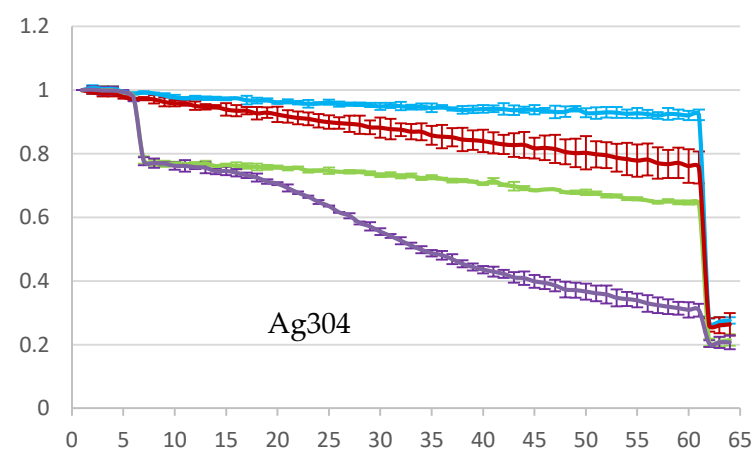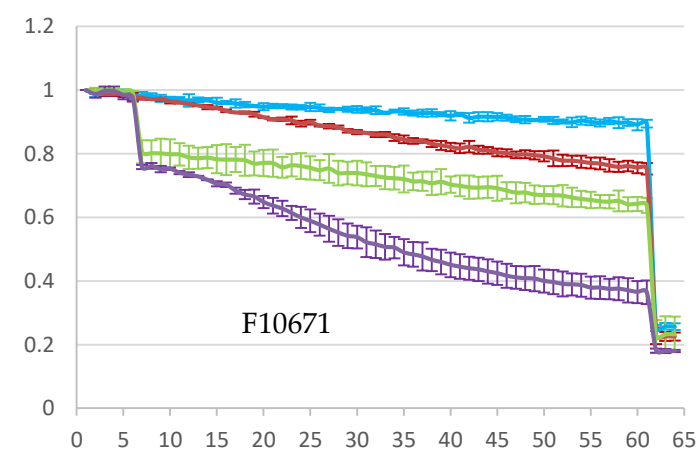

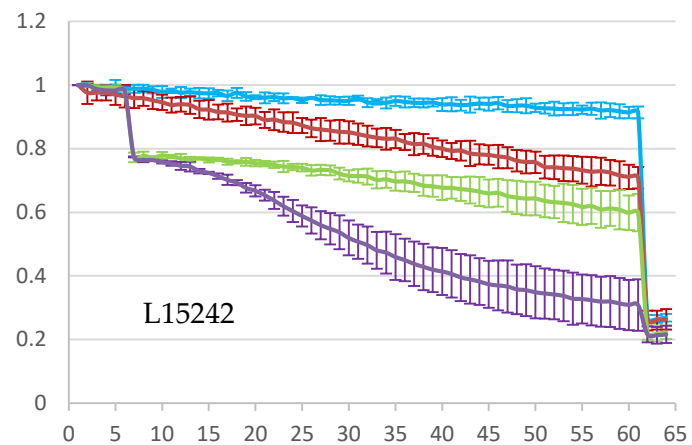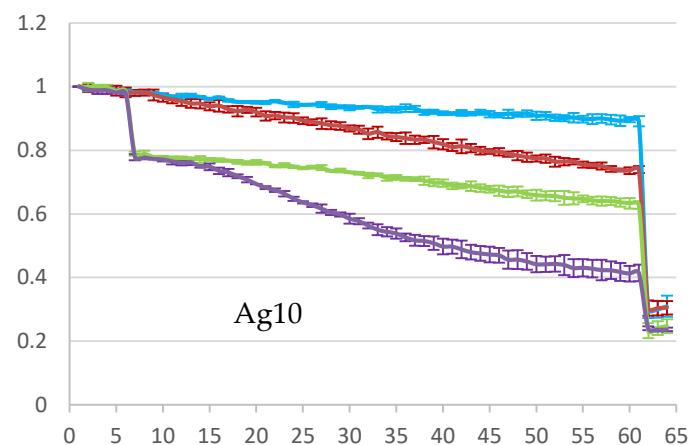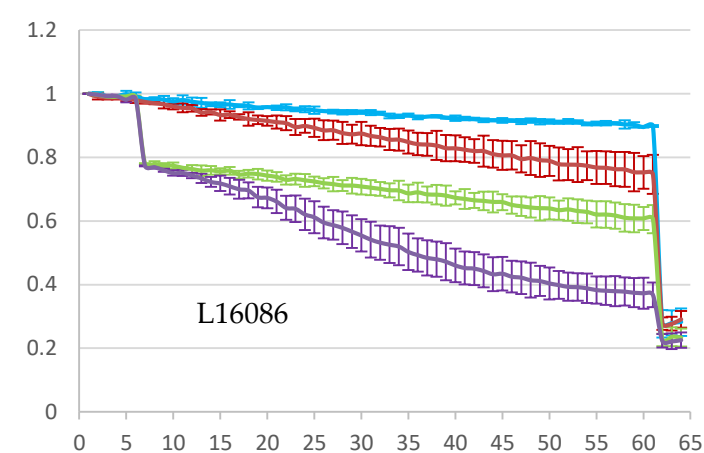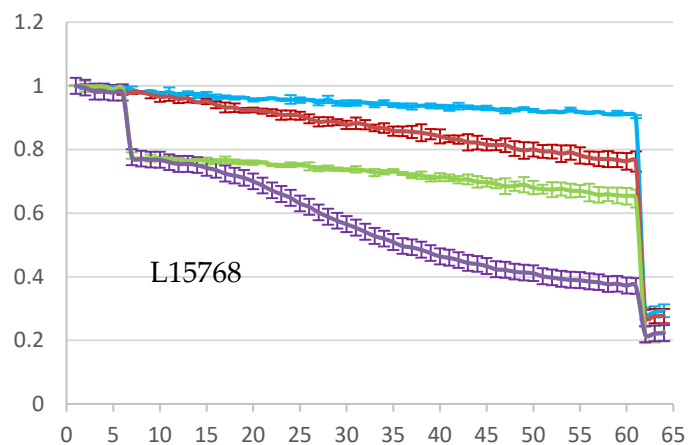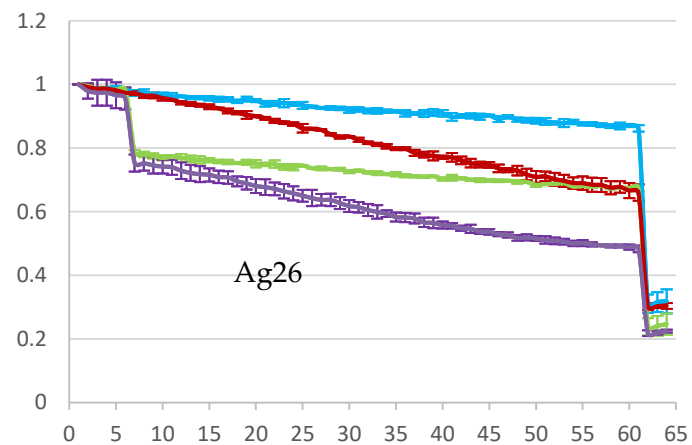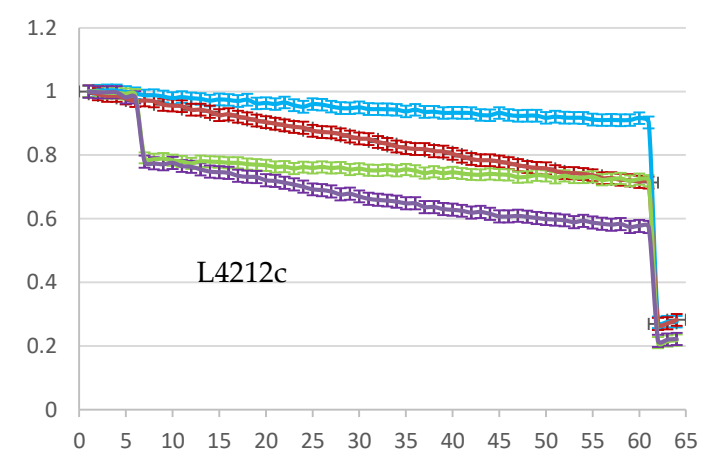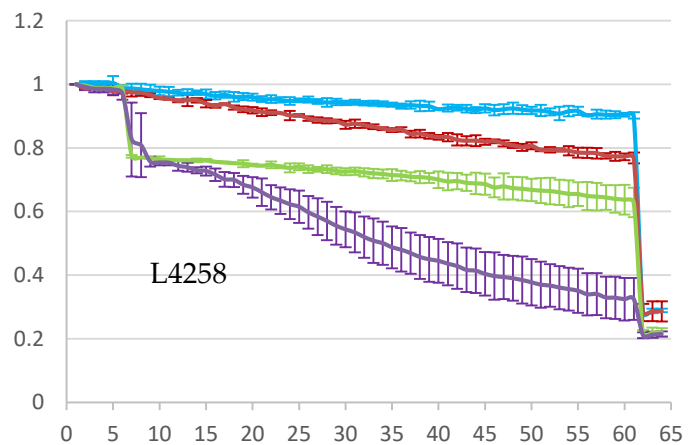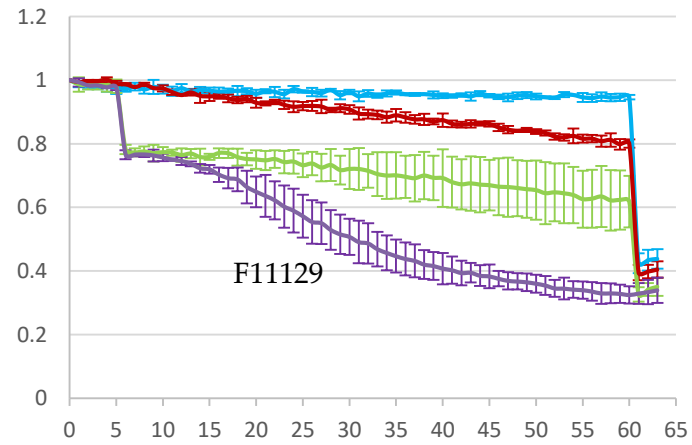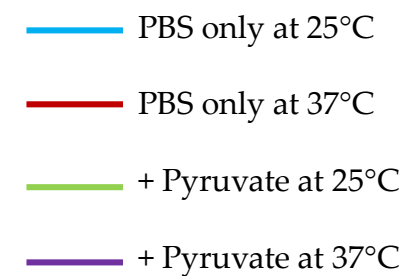

Supplement: Supplementary file 1 [file animals-11-02449-s001.zip › FigS1_MAGA.pdf]

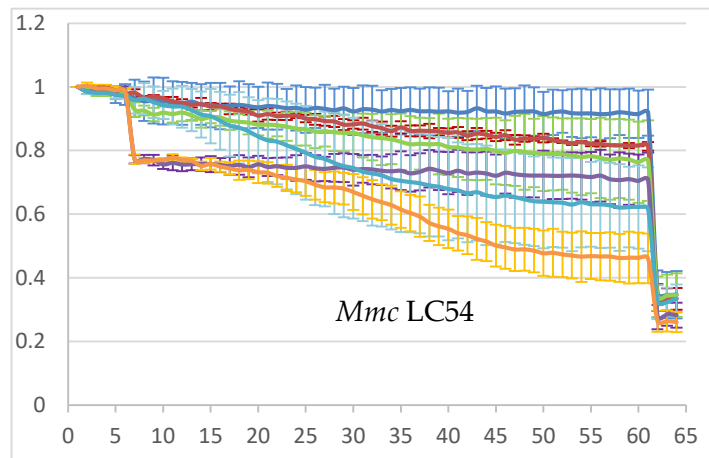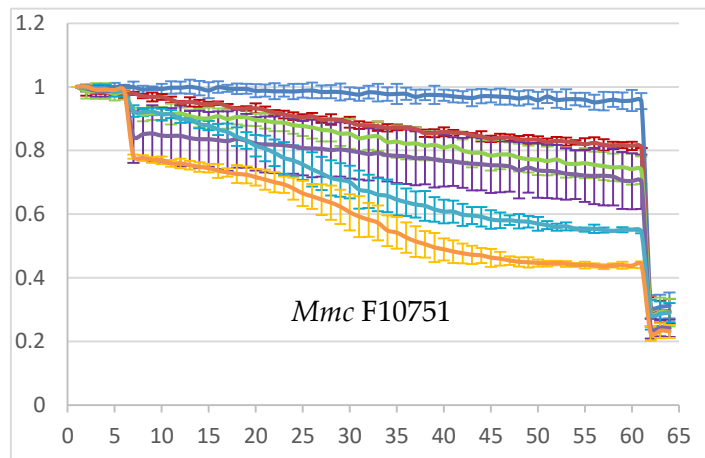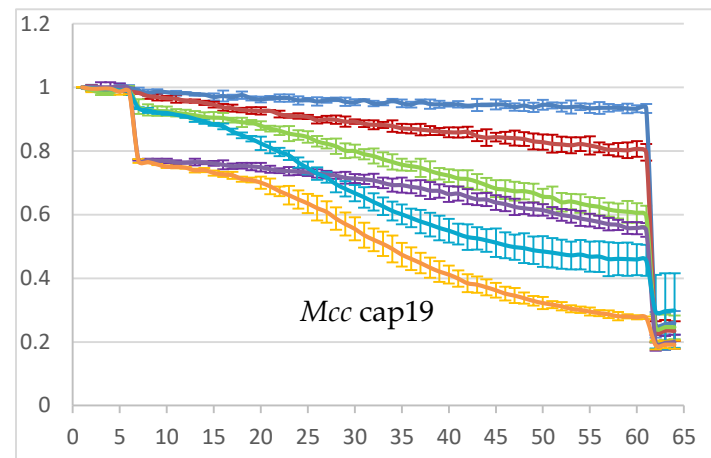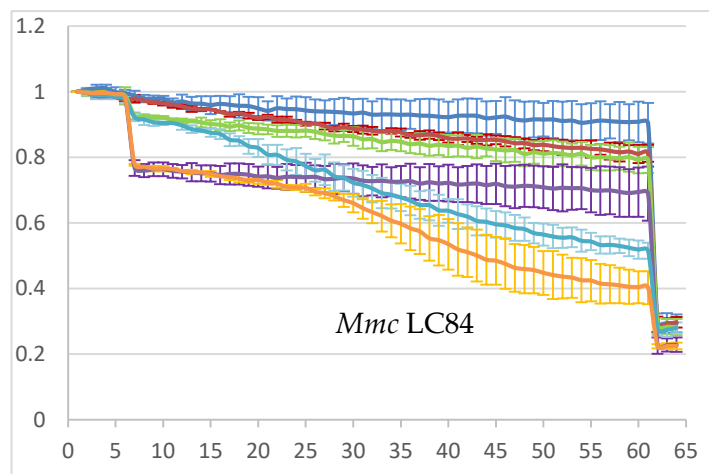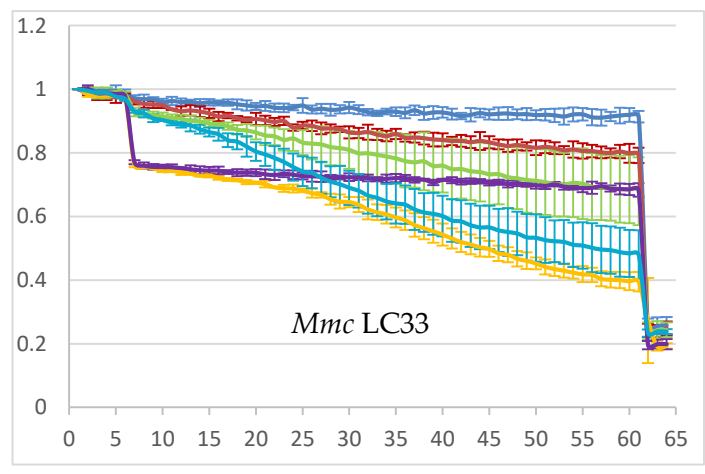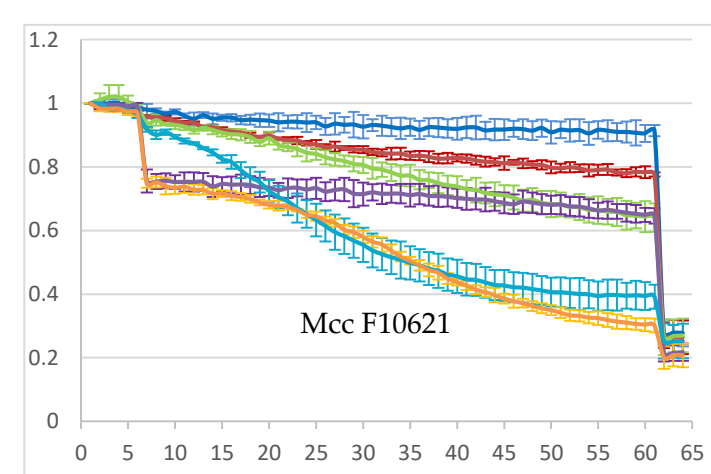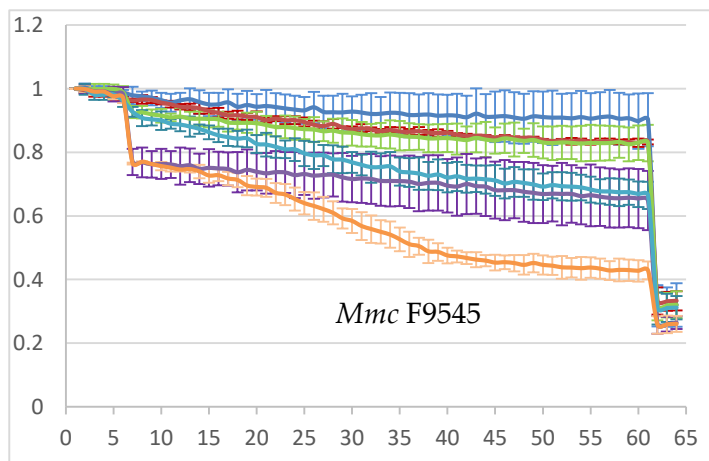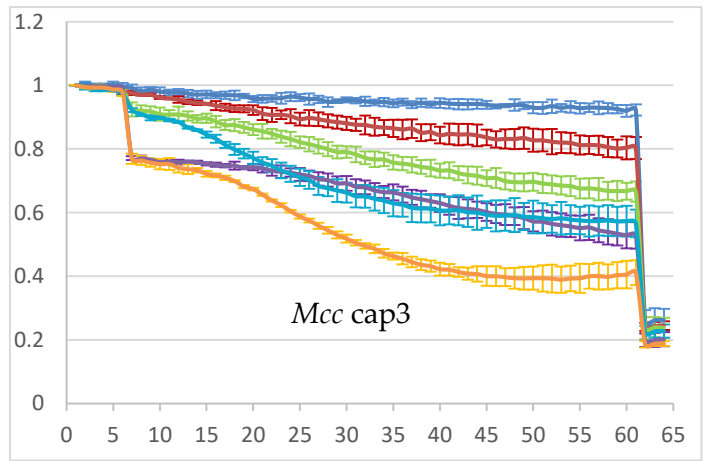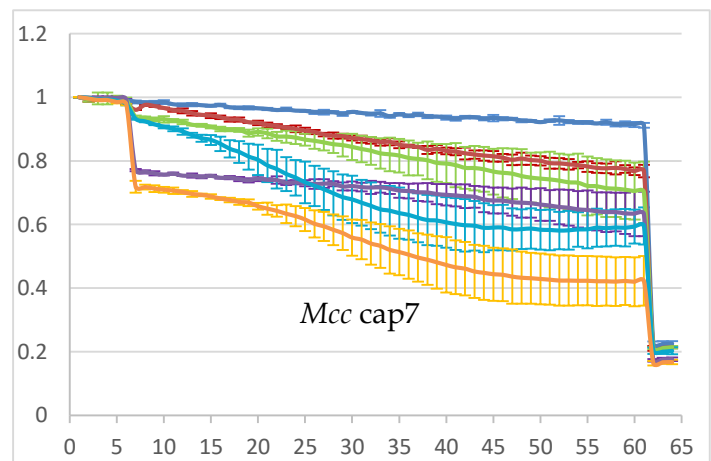

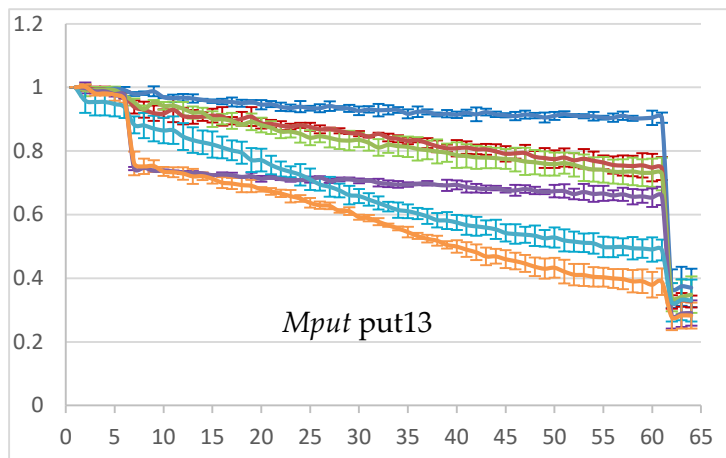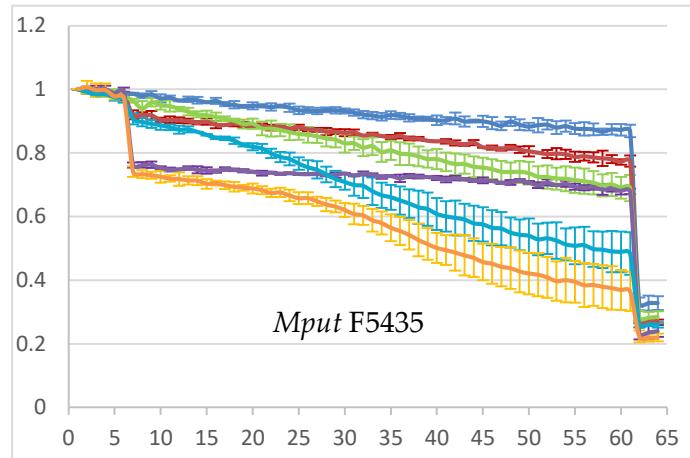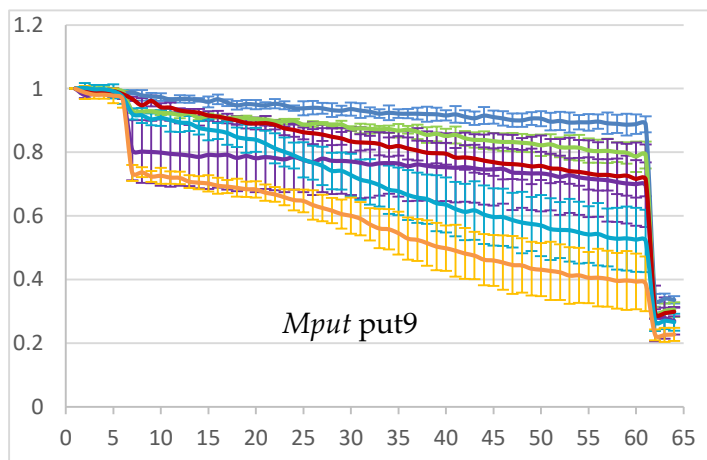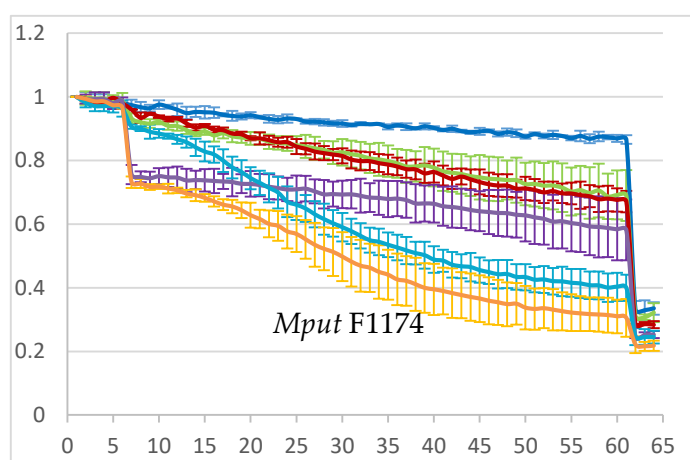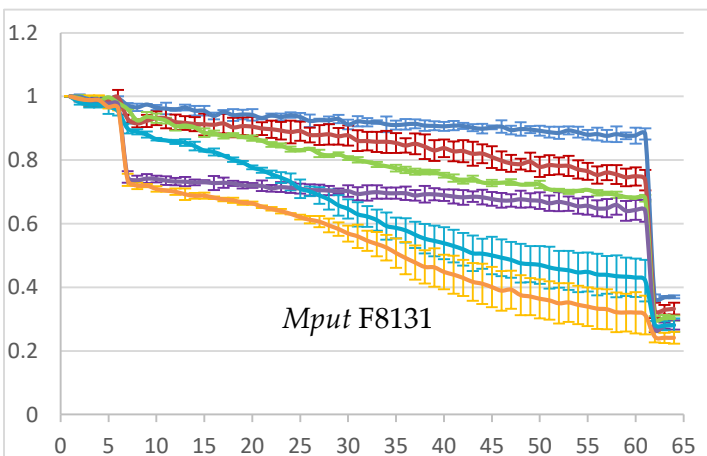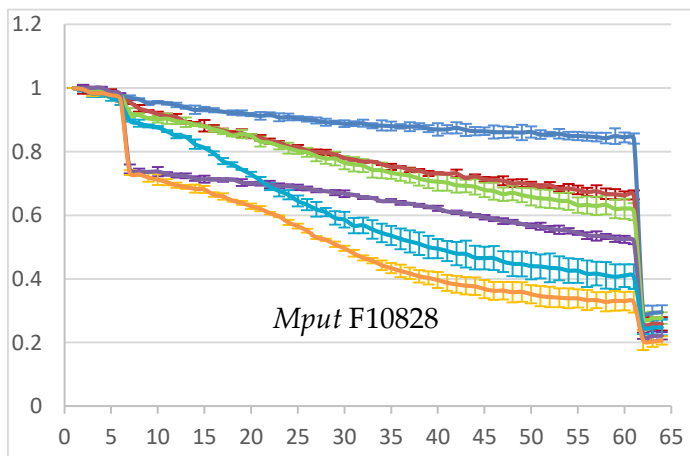

- PBS only at 25°C
- PBS only at 37°C
- + Pyruvate at 25°C
- + Pyruvate at 37°C
- + Glucose at 25°C
- + Glucose at 37°C

Supplement: Supplementary file 1 [file animals-11-02449-s001.zip › FigS2_MYC.pdf]
